# Development and Validation of a Behavioural Index for Adaptation to High Summer Temperatures among Urban Dwellers

**DOI:** 10.3390/ijerph14070820

**Published:** 2017-07-21

**Authors:** Pierre Valois, Denis Talbot, Maxime Caron, Marie-Pier Carrier, Alexandre J. S. Morin, Jean-Sébastien Renaud, Johann Jacob, Pierre Gosselin

**Affiliations:** 1 Faculty of Education, Université Laval, Québec, QC G1V0A6, Canada; maxime.caron@fse.ulaval.ca (M.C.); marie-pier.carrier@fse.ulaval.ca (M.-P.C.); johann.jacob@fse.ulaval.ca (J.J.); 2 Faculty of Medicine, Université Laval, Québec, QC G1V0A6, Canada; denis.talbot@fmed.ulaval.ca (D.T.); jean-sebastien.renaud@fmed.ulaval.ca (J.-S.R.); pierre.gosselin@inspq.qc.ca (P.G.); 3 Populations Health and Optimal Health Practices, CHU of Québec—Université Laval Research Center, Québec, QC G1S4L8, Canada; 4 Department of Psychology, Concordia University, Montréal, QC H3B1R6, Canada; alexandre.morin@concordia.ca; 5 Institut National de la Santé Publique, Direction de la Santé Environnementale et de la Toxicologie, Québec, QC G1V5B3, Canada; 6 Consortium on Regional Climatology and Adaptation to Climate Change, Montréal, QC H3A1B9, Canada

**Keywords:** adaptation index, climate change, heat wave, preventive health behaviours

## Abstract

One of the consequences of climate change is the growing number of extreme weather events, including heat waves, which have substantial impacts on the health of populations. From a public health standpoint, it is vital to ensure that people can adapt to high heat, particularly in cities where heat islands abound. Identifying indicators to include in a parsimonious index would help better differentiate individuals who adapt well to heat from those who do not adapt as well. This study aimed at developing and validating a summer heat adaptation index for residents of the 10 largest cities in the province of Québec, Canada. A sample of 2000 adults in 2015 and 1030 adults in 2016 completed a telephone questionnaire addressing their adoption (or non-adoption) of behaviours recommended by public health agencies to protect themselves during periods of high temperature, and their perceptions of how high summer heat affects their mental and physical health. Item analysis, confirmatory factor analysis, multiple correspondence analysis, measurement invariance analyses and criterion-validity analyses were used to develop a 12-behaviour heat adaptation index for distinguishing between individuals who adapt well to high temperatures and those who do not adapt as well. The results indicated that the measurement and the factor structure of the index were invariant (equivalent) across the two independent samples of participants who completed the questionnaire at different times one year apart, an important prerequisite for unambiguous interpretation of index scores across groups and over time. The results also showed that individuals who perceived more adverse effects on their physical or mental health adopted more preventive behaviours during periods of high temperatures and humidity conditions compared to those who felt lesser or no effects. This study thus presents support for the validity of the index that could be used in future studies to monitor preventive behaviours adoption during summer periods of high temperature.

## 1. Introduction

One of the negative consequences of climate change is the growing number of extreme weather events such as devastating heat waves [1,2,3]. High summer heat conditions (i.e., heat waves) have substantial impacts on the health of populations [4,5,6], ranging from premature death to hospitalisations and visits to hospital emergency units [7,8,9,10,11], especially among the elderly [12]. Moreover, residents of large cities are at greater risk for health consequences due to the presence of abundant mineral surfaces, lack of vegetation, and heat production sources that create the conditions for urban heat islands [13,14,15,16,17,18]. 

From a public health standpoint, it is vital to ensure that urban populations can adapt to summer heat waves, as the implementation of preventive measures have shown to reduce the occurrence of adverse effects during extreme heat [19]. The IPCC defines adaptation as an “adjustment in natural or human systems in response to actual or expected climatic stimuli or their effects, which moderates harm or exploits beneficial opportunities” [20]. This adjustment is happening through the conception and implementation of “policies, measures and strategies designed to reduce climate change impacts and support resilience [21,22]”, e.g., heath-health impacts [23]. Humans and society also adapt by building their adaptive capacity [24], which refers to their ability to adjust to potential damage, to take advantage of opportunities, or to respond to consequences [25].

Apart from physiological adaptation (where through short-term acclimatisation individuals maintain performance across a range of environmental conditions [20]), infrastructure, technological and behavioural adaptation are the main types of climate change adaptation related to heat [26]. Infrastructure adaptation is linked to reducing the risks to physical and organisational structures that are major societal investments [27], e.g., healthcare systems, roads, and communication systems [26]. Technological adaptation refers to retrofitting and replacing existing technologies to adjust to change [20]. Finally, behavioural adaptation refers to actions that households (individuals), communities, and populations can take to avoid or reduce a climate-change impact [20]. Depending on the timing, adaptation can be anticipatory or reactive, and depending on the level of planning involved, it can be planned or autonomous [2]. The focus of our study will be on behavioural adaptation.

There are many ways for individuals to adapt to heat waves, such as closing the curtains of the house when the sun shines, wearing light clothes, staying indoors, drinking more fluids [28,29] or using fans [30]. Such a wide range of behaviours is difficult to track over time. Identifying behavioural indicators to include in an index would help better distinguish individuals who adapt well to heat from those who do not adapt as well. The validation of such an index constitutes the first phase of a larger research project aiming, in phase 2, to identify the determinants of the general population’s heat adaptive behaviours based on a variety of theoretical frameworks (e.g., the theory of planned behaviour, Ajzen [31]; the health belief model, Rosenstock [32]). Then, in phase 3, this project aims to design and test an effective behaviour change strategy. The development of a reliable and valid measure of adaptation to heat waves in the general population is an essential preliminary step to giving credibility to conclusions from studies that will be conducted in phases 2 and 3. Moreover, the validity of this index has to be tested and established before it is used in governmental monitoring systems (e.g., to compare regions or age groups over time) to monitor the evolution of adaptive behaviours during heat waves.

Indexes are well recognized as being effective for describing complex issues that may be difficult to capture (e.g., vulnerability, poverty) and for reducing the set of relevant indicators or behaviours without losing information [33]. There are quite a few examples of indexes designed to assess vulnerability to climate change: e.g., the Global Vulnerability Assessment (GVA) and the GVA1 [34]; the Dynamic Interactive Vulnerability Assessment (DIVA) [35]; the Geographic Information Systems (GIS) [36]. These indexes and indicators mainly take into account levels of exposure, sensitivity, risk and adaptive capacity, as well as different data including in geographic, physical, social, economic or political domains. Therefore, they are useful to identify the most vulnerable populations and sectors and prioritize the needs with regards to adaptation, as well as to assess the performance of adaptation initiatives [37].

Because these indicators measure results to be achieved by the adaptation process on the longer run, on much larger perspectives (ecosystem, region, country, etc.), these metrics underplay the individual dimension of adaptation. In other work, these indicators do not really target any short and medium term adaptation behaviours. They rather stress that successful adaptation will be measured by how well different interventions will contribute to effectively reducing vulnerability and building resilience on environmental elements. While it is true that adaptation interventions seek to have a contribution on that level, it is likely to come in the longer term. Results on a shorter term, visible at the individual level, should also be visible and easier to monitor. Our research makes a significant contribution on that front. The development and validation of an index of adaptation to high temperatures will allow the measurement, as well as an assessment of the degree of progress towards reducing elements of vulnerabilities by targeting behaviours that are easily observable at the individual level.

Another problem pertaining to the measurement of progress towards adaptation lies in the difficulty to theoretically apprehend and to conceptualize the numerous definitions of adaptation [38]. Vague or undefined concepts and their various corollaries, which in some cases refer to similar or conversely similar ideas [39,40], as well as the inconsistency of what constitutes adaptation [41,42] are symptomatic of a lack of consensus which makes it difficult to establish metrics shared by a large number of actors [37]. Therefore, our contribution aims to identify a group of specific behaviours and create an index for recognizing, in various contexts, individuals who adapt to heat.

To our knowledge, only one study [43] has developed and validated an index of adaptation to heat waves (hereafter referred to heat adaptation index). Building on this previous work, our contribution will address three shortcomings. First, this index was developed for one specific population, that is, people living in very deprived areas. This is not compatible with the goal of this study, which consists in developing an index of adaptation to high temperatures that can be applied to the general population as part of governmental monitoring systems. Second, although the index underwent item analyses and tests of reliability and validity, a factor analysis was not conducted to test its unidimensionality (i.e., to ascertain that a single latent trait accounted for all the common variance among the behaviours composing the heat adaptation index). Third, this study did not examine whether the underlying factor structure of the index could be generalized to different samples of participants and over time. If the underlying factor is qualitatively different for distinct samples of people over time (e.g., comparisons of adaptation to heat each year on independent samples of people), mean differences and other comparisons are likely to be invalid. In other words, as suggested by Morin et al. [44], unless the index is measuring the same construct and is operating in the same way over time and for different groups of people, it may not be valid for determining mean differences between various subsamples taken from the general population over time. Consequently, a lack of measurement invariance of the index could lead to rather serious consequences, including inaccurate assessments of the evolution of the adaptive behaviours during heat waves over the years and the real impact of a behaviour change strategy on the people’s motivation to adopt adaptive behaviours during heat waves.

With these limitations in mind, our goal in this study was to develop and investigate the reliability, validity and measurement invariance of an index of adaptation to high temperatures that could be applied to the general population to determine whether urban dwellers adopt (or not) appropriate heat adaptive behaviours as recommended by public health agencies and the literature.

## 2. Materials and Methods 

### 2.1. Participants

To develop the heat adaptation index, we conducted two studies, the first in 2015 and the second in 2016. In both studies, we used a stratified sample of adult residents from the 10 largest cities in the province of Québec, Canada. These cities are located in a humid continental temperate climate zone [45]. Both samples were stratified by city and by material deprivation of the census dissemination areas (DAs). DAs were classified as advantaged, neither advantaged nor disadvantaged, or disadvantaged, based on census data, such as income level, education level, and employment status [46].

A two-stage design, similar to that proposed by Vallée et al. [47], was used to draw a sample that was representative of the 10 retained cities and of the deprivation levels of the DAs. In the first stage, we sampled a given number of DAs for each deprivation level of each city. The aim was to select a sample of DAs that was representative of the heterogeneity of all the DAs from which the sample was taken. More precisely, the subset of DAs sampled for a given deprivation level in a specific city needed to correspond to the set of all DAs coming from this specific deprivation level in this specific city with respect to the distribution of sex and age because existing literature outlines that these distributions influence adaptation to heat [48,49,50,51,52].

To determine whether the sampled DAs were sufficiently similar to the set of all DAs from a given deprivation level in a specific city, we computed, for sex and age, the ratio of the root-mean-square error (RMSE) in the sampled DAs over the variance of all DAs, following Vallée’s [47] method. If the ratio was close enough to 1, the sampled DAs were retained. Otherwise, other sets of DAs were randomly sampled until one meeting the correspondence criterion was obtained. In the second stage of the sampling, participants were randomly selected within the retained DAs for each of the 10 cities. The number of subjects sampled per DA was computed using Kish’s over-sampling method [53,54] with I = 0.7. This method allocates the total number of subjects to be sampled among DAs by compromising between proportional allocation (I = 1) and uniform allocation (I = 0). The Kish method was chosen to ensure that a minimum of 30 individuals in the 2015 study and 20 in the 2016 study were sampled for each deprivation level in each city, while keeping the sampling plan as close to proportional allocation as possible. All respondents were aged 18 years or older and spoke either French or English.

A total of 2000 individuals (1099 women, 55.0%; 901 men, 45.0%) were surveyed by a polling firm between 28 October and 14 December 2015, and another 1030 (626 women, 60.8%; 404 men, 39.2%), between 14 November and 13 December 2016. The response rates were 11.1% and 14.3%, respectively. A total of 29,645 telephone numbers were used for data collection. In the absence of contact at the first attempt, the interviewers made a maximum of 9 reminders (for a total of 10 attempts) at various times before rejecting the telephone number. The firm called only land lines (no mobile numbers). A list of addresses retrieved from “Adresses Québec” (a database providing information on all addresses throughout Québec) was sent to the polling firm. Its staff matched these addresses to phone numbers. No random-digit dialling procedure was used. The mean age of participants from the 2015 sample was 49.1 years (SD = 16.6). For 42.2% of the participants, the highest educational level obtained was a university degree; 31.6% reported an annual net income of 40,000 CAD or less, 33.2% an income of 40,001–80,000 CAD, and 35.2% an income of 80,001 CAD or more. The mean age of participants from the 2015 sample was 50.5 years (SD = 15.3). For 43.8% of the participants, the highest educational level obtained was a university degree; 31.2% reported an annual net income of 40,000 CAD or less, 33.7% an income of 40,001–80,000 CAD, and 35.1% an income of 80,001 CAD or more.

Our target population differ in several respects from that of Bélanger, Abdous, Gosselin and Valois [43], including education levels and more importantly, average household incomes. For instance, Bélanger, Abdous, Gosselin and Valois [43] noted that 55% of respondents did not have a post-secondary education, versus 45% in our study. An even greater gap was found in terms of income: 73% of their sample had a household income under 30,000 CAD, with 43% under 15,000 CAD, versus only 22% and 11%, respectively, in our sample.

Data were collected using a phone questionnaire that was pretested among 30 individuals. The questionnaire was developed based on adaptive behaviours recommended by public health agencies and the literature [55], and was inspired by the questionnaire developed by Bélanger, Abdous, Gosselin and Valois [43]. The data from the 2015 sample were used to test the reliability and validity of the index as well as its factor structure. The data from the 2016 sample were used to examine the measurement invariance of the index for different samples of people over time. No unusual heat waves occurred in either year.

### 2.2. Index of Adaptation to High Temperatures

To develop the heat adaptation index, 18 adaptive behaviours were initially selected and measured. This 18-item questionnaire is based on psychometric analyses performed by Bélanger, Abdous, Gosselin and Valois [43] of 90 adaptation items selected from a review of the literature on health and recommendations from public health agencies [56,57]. Several of these items were not considered in the creation of the heat adaptation index for various reasons, for instance a high correlation between two questions (for more details, see the online resource of the Bélanger and co-workers’ paper [43]). However, it also happened that two highly correlated questions were combined (e.g., swimming in a public or private pool). Finally, each adaptation behaviour includes more than one variable (or question). For example, one of the adaptation behaviours (i.e., drinking beverages to cool off when it is very hot and humid in summer) was assessed through a combination of one behavioural variable (main beverage consumed) and two non-behavioural variables (e.g., number of drinks per day). Similarly, the use of air-conditioning at home was assessed through a combination of two questions: do the households have access to air-conditioning at home; and if they have it, do they use it during heat waves. In the event that the households do not have air-conditioning, “no” was assigned for the use of air-conditioning.

The respondents were asked “When it is very hot and very humid in the summer, do you”: (1) swim in a private pool; (2) keep a list of emergency phone numbers on hand; (3) stay home during heat waves; (4) use air-conditioning during heat waves (this question was preceded by a question asking if the participants have or not an air-conditioning); (5) cover your head in strong sunlight; (6) take showers or baths more often than usual; (7) sponge or spray your face or neck with cool water; (8) consume frozen foods to cool down; (9) shut off the computer when not in use to reduce heat sources at home; (10) use the dryer less to reduce heat sources at home; (11) use the stove less to reduce heat sources at home; (12) use the balcony to cool off in the evening; (13) use the yard to cool off in the evening; (14) swim in a public pool, lake, or river to cool off; (15) spend time in air-conditioned places outside the home to cool off; (16) adopt preventive behaviours according to weather bulletins in the media or on the Internet; (17) use window shades to block strong sunlight and keep the home cool; and (18) drink mainly plain water to cool down. Respondents indicated how frequently they adopted each adaptive behaviour using a five-point ordinal scale ranging from “I never do this” to “I always do this.” The answers were then grouped into two categories: behaviours that people adopt (I usually do this; I always do this) and behaviours that people do not adopt (I sometimes do this; I rarely do this; I never do this). We merged the “I sometimes do this” response option with the “I rarely do this” and “I never do this” response options on the basis of statistical analyses showing a relatively low response variability for the “I rarely do this” and “I never do this” options, suggesting that true differentiation between adaptation and non-adaptation occurred at the higher end of the response scale. Furthermore, these additional analyses also showed that considering the “I sometimes do this” response option in the “behaviours that people adopt” category or in the “behaviours that people do not adopt” category did not change our results. Respondents who did not have a computer, clothes dryer or oven and respondents who had a yard or balcony were considered to do the right behaviour. 

### 2.3. Statistical Analyses

The individuals in the sample were reweighted using information concerning: (1) the interaction between deprivation level and city; (2) sex; (3) age; and (4) education [58]. More specifically, as a calibration estimator, we used a blocking approach called the raking ratio estimation [59]. This method improves the estimation of the variable of interest (adaptation to high temperatures) because the variance of the estimator is reduced when the auxiliary variables are correlated with the variable of interest. Because there were some missing values for age and education level, we imputed the missing data for these variables using predictive mean matching [60].

First, to assess the psychometric qualities of the adaptation index, we performed an item analysis, using Samejima’s graded response model [61]. The objective of this item analysis was to assess the performance of the items according to a certain number of psychometric parameters (e.g., the ability to distinguish between individuals who adapt well to heat and those who do not adapt as well) and to determine which items to retain in the final measure. The discriminant parameter could thus be conceived as a description of the association between the item and the measured construct, because the higher the discrimination index for an item, the better that item can distinguish between high and low adapters. Baker [62] proposes the following classification to interpret discrimination ability: (a) very poor: 0.34 or less; (b) poor: 0.35–0.64; (c) moderate: 0.65–1.34; (d) good: 1.35–1.69; and (e) very good: 1.70 or higher. 

Second, we conducted a confirmatory factor analysis (CFA) to assess the dimensionality of the heat adaptation index. We tested a parsimonious model that included all the behaviours within a single construct representing adaptation to high temperatures. We then assessed the compatibility of the empirical data with the hypothetical measurement model using various fit indices described below.

Third, a multiple correspondence analysis (MCA) was also conducted. MCA is a data reduction procedure [63] that is frequently performed when constructing composite indices [43,64,65]. The percentage of inertia computed using Greenacres [66] method served to counter-validate the results of the CFA regarding the unidimensionality of the heat adaptation index. The MCA results were also interpreted in terms of the contribution of the measured behaviours to the factorial dimensions obtained by MCA.

Fourth, we assessed the measurement invariance (equivalence) of the index across two independent samples of participants who completed the questionnaire measuring their heat adaptive behaviours at different times one year apart. This invariance is a necessary condition for unambiguous interpretation of index mean differences over time [44,67]. Thus, using the data collected in 2015 and 2016, we performed measurement invariance tests to evaluate the extent to which measurement properties of the adaptation index generalize over time to different samples of people. These tests were performed in the following sequence, adapted to the binary nature of the items [67,68]. First, a model with no invariance of any parameters, also referred to as the configural invariance model, was estimated. Typically, tests of measurement invariance then separate tests of weak invariance (invariance of the factor loadings) from tests of strong invariance (invariance of the factor loadings and item thresholds), which is not possible to do when using binary items [68]. Thus, we directly tested the strong invariance of the model by constraining the factor loadings and items’ thresholds to equality across groups. A violation of the invariance of factor loadings suggest that the index does not assess the same constructs across groups, whereas a violation of the invariance of the items’ thresholds suggest that participants’ responses on the items differ systematically across groups irrespective of their true score on the underlying construct. Third, we tested the strict invariance of the model by constraining the factor loadings, items’ thresholds, and items’ uniquenesses to equality across groups. A violation of the invariance of the items’ uniquenesses would indicate that the measurement errors of these items are non-equivalent across groups. In a fourth and fifth steps, we finally tested the invariance of the latent variance and latent mean of the estimated factors. These last two tests are not indicative of measurement bias, but rather of specific group-based differences in terms of within-group variability and means.

Whereas weak invariance is a prerequisite for meaningful comparisons of relations between latent variables across groups, strong invariance is a prerequisite for latent mean comparisons between groups [69]. However, given that latent variable models include a natural control for measurement errors, strict invariance is not a requirement for group-based comparisons based on latent variable models (as in this study), although there are critical for comparisons based on manifest scale scores, such as those planned for the future use of this index. In each step of the sequence, the preceding model served as a referent (e.g., Guay et al. [70]; Morin et al. [71]).

The fit of all models was evaluated using various indexes as operationalized in Mplus 7.4 [68] in conjunction with the weighted least squares means and variance adjusted (WLSMV) estimator [72,73]. Given the known oversensitivity of the chi-square to sample size, minor deviations from normality, and minor model misspecifications, model fit is usually assessed with sample-size-independent fit indices which, in this study, were the comparative fit index (CFI), the Tucker-Lewis index (TLI), and the root mean squared error of approximation (RMSEA). According to conventional rules of thumb [72,74], acceptable model fit is indicated by CFI and TLI values greater than or equal to 0.90 and less than 0.95, and excellent model fit is indicated by CFI and TLI values greater than or equal to 0.95. Moreover, RMSEA values between 0.05 and 0.08 indicate an adequate model fit, while RMSEA values less than or equal to 0.05 indicate an excellent model fit. Regarding model comparisons, Cheung and Rensvold [75] as well as Chen [76] note that a CFI or TLI decrease of 0.01 or less and a RMSEA increase of 0.015 or less from one model to the next can be taken to indicate that the invariance hypothesis should not be rejected.

### 2.4. Criterion-Related Validity of the Index

Once the psychometric properties of the adaptation index were confirmed, we conducted a criterion validity analysis. The essential function of the criterion validity analysis is to define the relationship between test results (here, the score on the heat-adaptation index) and another criterion considered to be an indicator of the construct to study [77]. In this current study, this indicator corresponds to the self-reported adverse health impacts. The validity of self-reported versus medical-based diagnoses and behaviours has been well established over time, in several countries and data collection methods, especially as a tool for predicting future risks and as an epidemiologic survey tool for prevention and public health actions [43,78,79,80,81].

More specifically, the respondents were asked the two following questions: “Would you say that your physical health is adversely affected when it is very hot and humid in the summer?” and “Would you say that your mental health is adversely affected when it is very hot and humid in the summer?” Both questions were rated on 4-point ordinal scales: “Not at all”, “Slightly”, “Moderately”, and “Very much”. Individuals who reported feeling moderate or severe adverse physical or mental effects on their health were considered an at-risk group, and those who reported no or few such effects were considered a lower risk comparison group. The prevalence of perceived adverse effects was compared between adaptation levels using the odds ratio to test the criterion-related validity of the index.

As described in the Model of Private Proactive Adaptation to Climate Change (MPPACC) suggested by Grothmann and Patt [82], risk perception is an important psychological dimension of adaptation to climate change. Moreover, some studies have established that risk perception was positively linked to adaptation behaviours [83,84,85]. More specifically, it has been observed that there is a larger proportion of individuals perceiving moderate or severe adverse physical or mental effects on their health who adapt well to heat than of individuals perceiving no or slightly adverse physical or mental effects on their health [55,86]. Thus, we also expected to observe such an association in our study.

## 3. Results

### 3.1. Item Analysis

The results of the item analysis using the Excel add-in EIRT [87] revealed that the adaptation index had good reliability: most of the 18 adaptive behaviours appeared to properly measure the heat adaptation construct. Only one behaviour, “use air-conditioning during heat waves”, seemed problematic, with a discrimination index approaching zero (−0.057; see Table 1). This item was therefore removed from the index. Interestingly, this low discrimination ability was not due to the fact that almost all respondents reported using air-conditioning (i.e., a low response variability): 32.63% of respondents did not use air-conditioning during heat waves compared to 67.37% who did. This low discrimination power could rather be explained by the fact that individuals might want to use air-conditioning but be unable to due to the high cost involved or other reasons. The ANOVA result supports this hypothesis because respondents with incomes below 40,000 CAD reported using less air-conditioning compared to respondents with incomes above 40,000 CAD: F(3, 1678) = 7.63, *p* < 0.01.

### 3.2. Confirmatory Factor Analysis

**Figure 1:**
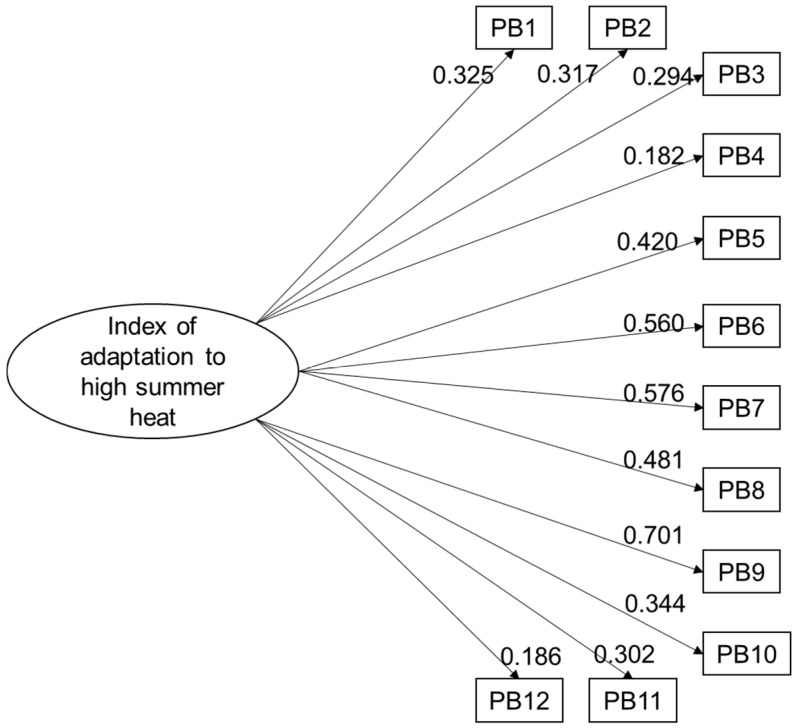
Results for the final model tested with a confirmatory factor analysis. Legend: PB1. Cover you head in strong sunlight; PB2. Take showers or baths more often than usual; PB3. Drink mainly plain water to cool down; PB4. Swim in a public or private place during high heat and humidity; PB5. Adopt behaviours according to weather bulletins; PB6. Use window shades to block strong sunlight and keep the home cool; PB7. Use the dryer less; PB8. Shut off the computer when not in use; PB9. Use the stove less; PB10. Spend time in air-conditioned places outside the home; PB11. Use the balcony in the evening; PB12. Keep a list of emergency phone numbers on hand.

The factor validity of the index was then tested by determining whether the adaptive behaviours corresponded to a single construct: adaptation to high temperatures. The results showed a poor fit of the data with the theoretical model that included the 17 adaptive behaviours retained after the item analysis (CFI = 0.631, TLI = 0.558, RMSEA = 0.050). The modification parameter estimates and indices provided in Mplus were therefore used to identify potentially problematic indicators (i.e., loadings < 0.30; substantial correlate residual). Table 2 presents the revised model after five problematic behaviours were sequentially removed from the index. The results (Table 2, last line) show that the revised model had an adequate level of fit to the data (CFI = 0.921, TLI = 0.903, RMSEA = 0.023). To further improve the index validity, we tested the revised model by combining certain behaviours that were not retained in the previous steps but were correlated and complementary to the behaviours that were retained to make up the index. For example, we created a unique variable that combined the behaviours “swim in a public place” and “swim in a private place” to obtain a more general behaviour called “Swim in a public or private place during high heat and humidity”. The results (not shown) indicated that only one of the four combinations improved the quality of the index: “Swim in a public or private place”. The final model therefore comprised 12 behaviours (including one combined behaviour) and adequate level of fit to the data (CFI = 0.926, TLI = 0.909, RMSEA = 0.022; see Figure 1).

### 3.3. Multiple Correspondence Analysis

A multiple correspondence analysis was then performed on the 12 retained indicators. The results revealed that the total inertia explained by the first and second dimensions were respectively 83.8% and 1.2%, indicating, as did the CFA results, that the index is unidimensional. 

**Figure 2:**
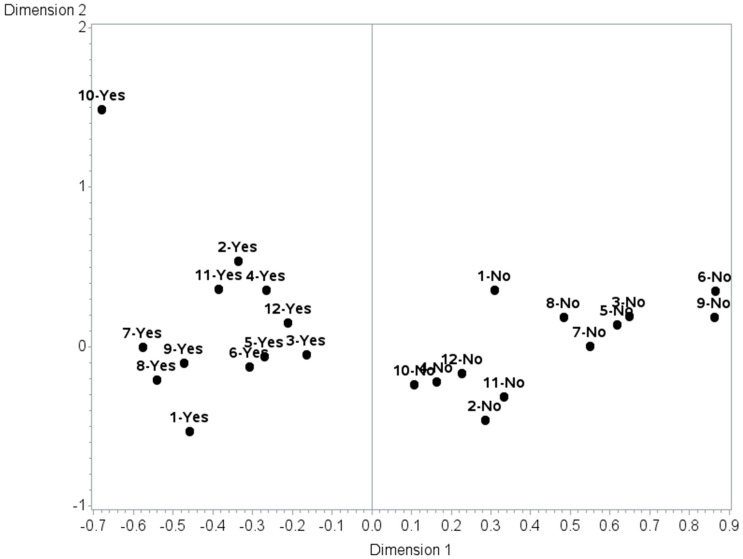
Projection of the active variables in the MCA. Legend: For all variables, “Yes” and “No” represents responses indicating that people adopt or do not adopt, respectively, the following behaviours: 1. Cover your head in strong sunlight; 2. Take showers or baths more often than usual; 3. Drink mainly plain water to cool down; 4. Swim in a public or private place during high heat and humidity; 5. Adopt behaviours according to weather bulletins; 6. Use window shades to block strong sunlight and keep the home cool; 7. Use the dryer less; 8. Shut off the computer when not in use; 9. Use the stove less; 10. Spend time in air-conditioned places outside the home; 11. Use the balcony in the evening; 12. Keep a list of emergency phone numbers on hand.

The projected coordinates of the active variables (i.e., the 12 behaviours) show that all the responses indicating that people adopt the behaviours (i.e., I usually or always adopt the behaviour) are situated at the left side of the plot, with all the responses indicating that people do not adopt the behaviours (i.e., I never adopt the behaviour; I rarely adopt the behaviour; I sometimes adopt the behaviour) are situated at the right side of the plot (see Figure 2). The contribution of the different behaviours allows us to determine which ones are more associated with adaptation and which ones, with non-adaptation. The results show that, in terms of their contribution, the behaviours that people adopt are more strongly associated with the construct of adaptation to heat (data not shown). First comes “Use the clothes dryer less to reduce heat sources at home”, followed by “Use the stove less”. Next comes “Shut off the computer when not in use” and “Cover your head in strong sunlight”. Of the behaviours that people do not adopt, the most strongly associated with the construct of non-adaptation to heat is “using the stove less”, followed by “using window shades to block strong sunlight and keep the home cool”. “Using the dryer less” is the third most strongly associated with behaviours that people do not adopt.

### 3.4. Measurement Invariance

We tested the measurement invariance of the heat adaptation measurement model across the independent samples of participants who completed the survey measuring their heat adaptive behaviours in 2015 and 2016, respectively. The results from these tests are reported in Table 3. These results show that, throughout the full sequence of invariance tests, all of the increasingly restrictive models provided an adequate level of fit to the data, with CFI and TLI *>* 0.90 and RMSEA *<* 0.03. Moreover, no ∆CFI exceeded −0.01 and no ∆RMSEA exceeded +0.015, providing support for the strong, strict, latent variance and latent mean invariance across the groups of participants.

Starting from the model of latent variance invariance, we then used a method developed by Little et al. [88] and used by Litalien et al. [89] to conduct an ANOVA-like latent means comparison across groups of participants within a latent variable framework. The result, which is expressed as a between-group deviation in standard deviation units, showed that, on average, the participants surveyed in 2016 reported the same levels of preventive behaviour adoption during summer periods of high temperatures as the participants surveyed in 2015 (deviation = −0.10, *p* = 0.16). This conclusion is consistent with the previously reported result showing that the invariance of the latent means was supported.

### 3.5. Criterion-Related Validity of the Index

Adaptation scores were generated using the coordinates of the 12 index components of the multiple correspondence analysis. The scores ranged from −5 to +5 and displayed a quasi-normal distribution (for more details on how these scores were generated, see Greenacres [66]). The results suggest that 53.12% of the respondents adapted well to high heat (score < 0) versus 46.88% who did not adapt as well (score > 0). To test the criterion validity of the index, we calculated the prevalence of health impacts (at-risk group, lower risk group) according to the adaptation level as measured by the dichotomized index (adaptation and non-adaptation). The results (see Table 4) show that the index has good criterion validity. In fact, there is a larger proportion of individuals perceiving moderate or severe adverse physical or mental effects on their health who adapt well to heat than of individuals perceiving no or slightly adverse physical or mental effects on their health: the prevalence of health impacts is estimated at 45.77% for individuals who adapt well to heat and 38.11% for individuals who do not adapt as well (odds ratio = 1.37, *p* = 0.0011). As a sensitivity analysis, we tried using cut-offs of −0.16 and 0.11, which correspond to the smallest values on the plot representing the people’s responses indicating that they adopt or do not adopt the behaviours, respectively. Similar results were obtained.

## 4. Discussion

This study is a substantive-methodological synergy [90], bringing to bear strong evolving methods to develop and validate a new unidimensional index designed to measure heat adaptive behaviours in urban settings.

This validated index is parsimonious, as it is based on only 12 behaviours that enable individuals to lower their body temperature, protect themselves from sun exposure at home and outdoors, maintain a cool household temperature, and reduce heat sources within their houses. These behaviours are simple actions that are recommended by public health agencies to protect individuals against high heat conditions, and should consequently reduce the severity of adverse effects on mental and physical health. This study provides a valid tool to measure how well people are adapting to heat. The total inertia explained by the first dimension in the multiple correspondence analysis and the fit indices obtained in the confirmatory factorial analysis both support the unidimensionality of the index. More precisely, these results support the idea that a single latent trait was able to account for most of the variance shared among the behaviours included in this index. Furthermore, our results demonstrate the measurement invariance of the 12-item model across two independent samples of participants from the same population who completed the questionnaire measuring their heat adaptive behaviours at different times one year apart. These findings suggest that the results obtained from this index can be generalized to different samples of participants in the province of Québec (Canada) both now and into the future, as it is suitable for the culture and climate of Quebec. It therefore affords the prospect of meaningful comparisons of heat adaptation data over time regarding the level of preventive behaviours adopted during summer periods of high temperatures.

Thus, our findings have major implications for governmental and municipal heat wave planning frameworks. In fact, our results provide strong evidence that this index could be used in future surveys to monitor heat adaptation regularly in urban populations. This kind of monitoring over time could enable public health agencies to better identify protective measures to incorporate in health promotion campaigns. It should also enable better targeting of at-risk groups and groups that are not adapting as well. Based on the level of adoption of these various behaviours by different population sub-groups, interventions aiming to promote these behaviours will eventually be developed (e.g., advertising messages, training for medical personnel, and development of governmental documentation). 

Although our index may appear quite similar to that presented in another study by our research group [43], it differs substantively. First, our results show that our index can be applied not only to people living in very deprived areas, but also to the general population. The two populations differ in several respects, including education levels and more importantly, average household incomes. Consequently, it is not surprising that content analyses of the two indices indicate a reasonable item overlap (i.e., 10 heat adaptive behaviours common to both indices and six specific to one or the other). This finding is important and reinforces the necessity, when constructing an index, to refer to behaviours corresponding to the reality of the targeted population. To lend credence to this idea, it would be useful to conduct additional studies in different populations. 

However, consistent with the study by Bélanger, Abdous, Gosselin and Valois [43], home air-conditioning was not retained in the index based on the psychometric analyses. As noted by Bélanger, Abdous, Gosselin and Valois [43], this finding goes against what the literature generally shows, that is, the use of home air-conditioning is recognized as an effective way for individuals in poor health to adapt to summer heat waves. This does not mean that public health authorities should stop favouring the use of air-conditioning as a way to adapt to heat. It means only that, statistically, measuring this behaviour is less effective for recognizing people who are adapting to heat from those that are not adapting as well to heat. It is possible that the weak association observed between air-conditioning and the index is a statistical artefact, given the relatively high number of respondents (67.37%) in our study who said that they lived in housing equipped with air-conditioning and that they used it to deal with high summer heat. This result indicates that Québec residents are aware that home air-conditioning is an effective way to adapt to heat and, as such, explain why the inclusion of this item in the index did not help to improve its ability to discriminate respondents. Nevertheless, future studies focusing on the factor structure of heat adaptation could provide more insights into this particular behaviour.

Taken at face value, this is good news, because home air-conditioning is known to reduce deaths and illnesses during heat waves [91]. We might also suppose that individuals may express a desire to use air-conditioning to protect themselves against heat, but refrain from doing so because of the expenses related to the air-conditioning itself and its power usage, their dislike for cold air blowing on them, their perception that air-conditioning produces anthropogenic heat contributing to the urban heat-island effects [92,93], or the loud noise it emits [94,95,96]. Our results also indicate that people with lower incomes use less air-conditioning than those with higher incomes. Thus, it is possible that the financial barriers, along with the high running costs, could in part explain the weak association observed between air-conditioning and the index in this study. This does not diminish the importance of this behaviour (i.e., everyone should have access to it because it so effective), and, in our view, it would be advisable to add this indicator to monitoring systems that address adverse health effects.

Another contribution of this study is that our results support the complete measurement invariance of the proposed index across two independent samples of participants who completed the questionnaire measuring their heat adaptive behaviours at different times approximately one year apart. This invariance could not be demonstrated in our previous study because it included only a one-time measurement. The latter result is important because a lack of measurement invariance of the index could lead to rather serious consequences, including inaccurate evaluations of the evolution of the adaptive behaviours during heat waves over the years.

The results also show that there is a positive relation between perception of adverse effects on physical or mental health and the adoption of preventive behaviours to protect against summer periods of high temperatures. However, at this point, we cannot determine whether the adoption of all these behaviours has an impact on their perceived effectiveness. In future studies, it would be instructive to include a question to verify whether the most adaptive individuals, according to the index, are also those who perceive these behaviours as effective, and if these behaviours have already decreased their health impacts in their opinion.

Several limitations should be considered when interpreting these findings. First, all the data came from self-reported measures. Second, given the low response rates obtained, the samples cannot be considered representative of all urban dwellers. For example, we cannot reject the hypothesis that the sample obtained was composed mainly of individuals who were actually concerned about heat waves. Despite this limitation, individuals who agreed to participate in the study responded to the great majority of the questions. Third, to ensure that this index can be used across countries concerned with heat wave conditions, its measurement invariance in such countries must first be demonstrated in independent samples. Fourth, it would also be useful to test in the future the measurement invariance of the index across income status and educational levels. Fifth, respondents may have had recall bias given that the survey was undertaken in the cooler months and not in the hot season. Sixth, some of the items in the index may conflate the adaptive capacity (availability of the resource) with the adaptive behaviour itself. For example, if the respondent does not have a private pool, balcony, or yard, s/he cannot adopt this behaviour. Saying those people are non-adaptive seems to be inaccurate. It is also important in the future to distinguish between capacity and behaviour and test their relation in a predictive model of adaptation to heat waves. To achieve this goal, researchers will have to: (1) define these constructs theoretically; (2) test their psychometric properties; and (3) verify the link of causality between them. Furthermore, the criterion validity analysis conducted did not involve any assumption of causality or directionality. Future research should verify the link of causality between self-reported adverse health effects and adaptive behaviours. Finally, we believe that additional studies, considering a wider range of criterion measures of adaptation or non-adaptation, administered after the index itself, would be required in order to be able to identify a cut-off sore that is as precise as possible.

## 5. Conclusions

The purpose of this article was also to provide a substantive-methodological synergy of potential importance to future research in measurement to adaptation to climate change. The substantive focus was to improve the measurement of future indices regarding different hazards by using a strong, and evolving methodology based essentially on a measurement the ory (i.e., item response theory) and the construct of measurement invariance in order to evaluate the psychometric properties of indices. We think that the methodology we use could also be applied for the development of other indices (e.g., adaptation to flood and pollen allergy, and vulnerability to tick-borne disease).

Our goal in this study was to develop and investigate the reliability, validity and measurement invariance of an index of adaptation to high temperatures that could be applied to the general population to determine whether urban dwellers adopt appropriate heat adaptive behaviours as recommended by public health agencies and the literature. In conclusion, although additional tests of the measurement invariance of the index across different countries are needed, this study underscores its validity. Thus, researchers and public health agencies and professionals can already use the index to monitor the evolution of individuals’ adaptive behaviours during heat waves, in the opinion of the authors.

## Figures and Tables

**Table 1:** Discrimination indices for each adaptive behaviour.

Adaptive Behaviours	Discrimination Index	99% Confidence Interval
Cover your head in strong sunlight	0.512	(0.386–0.638)
Sponge or spray your face or neck with cool water	0.962	(0.790–1.134)
Take showers or baths more often than usual	0.753	(0.615–0.890)
Drink mainly plain water to cool down	0.473	(0.323–0.622)
Consume frozen foods to cool down	0.512	(0.382–0.641)
Swim in a public pool, lake, or river to cool off	0.591	(0.427–0.755)
Swim in a private pool to cool off	0.394	(0.261–0.526)
Adopt preventive behaviours according to weather bulletins in the media or on the Internet	0.856	(0.703–1.009)
Stay home during heat waves to avoid adverse health effects	0.441	(0.315–0.566)
Keep a list of emergency phone numbers on hand	0.314	(0.195–0.432)
Use air-conditioning during heat waves	–0.057	(–0.178–0.063)
Use window shades to block strong sunlight and keep the home cool	0.966	(0.803–1.128)
Use the dryer less to reduce heat sources at home	1.132	(0.972–1.293)
Shut off the computer when not in use to reduce heat sources at home	0.789	(0.651–0.928)
Use the stove less to reduce heat sources at home	1.359	(1.175–1.543)
Spend time in air-conditioned places outside the home to cool off	0.782	(0.589–0.974)
Use the balcony to cool off in the evening	0.731	(0.595–0.866)
Use the yard to cool off in the evening	0.641	(0.506–0.776)

**Table 2:** Indicators removed from the CFA model.

Removed Behaviours	Number of Behaviours Composing the Index	Reason	Model Fit			
			CFI ^a^	TLI ^b^	χ^2^/*df* ^c^	RMSEA ^d^
None: Initial model	17		0.631	0.578	5.99	0.050
Use the yard to cool off in the evening	16	Too high a relationship with “Use the balcony to cool off in the evening” (*r* = 0.67)	0.741	0.701	3.87	0.038
Sponge or spray your face or neck with cool water	15	Too high a relationship with “Take showers or baths more often than usual” (*r* = 0.49)	0.751	0.709	3.62	0.036
Swim in a private pool	14	Too high a relationship with “swim in a public pool” (*r* = 0.27)	0.812	0.778	3.17	0.033
Stay home during a heat wave	13	Too high a relationship with “Adopt preventive behaviours according to weather bulletins in the media or on the Internet” (*r* = 0.38)	0.876	0.851	2.47	0.027
Consume frozen foods to cool down	12	Does not appear to belong to the same theoretical construct as that measured by the other indicators. One possible explanation is that the respondents did not necessarily adopt this behaviour to combat heat.	0.921	0.903	2.02	0.023

^a^ CFI: Comparative Fit Index; ^b^ TLI: Tucker-Lewis Index; ^c^ χ^2^/*df*: Chi-square/degrees of freedom; ^d^ RMSEA: Root Mean Squared Error of Approximation.

**Table 3:** Goodness of fit indices of the models tested.

Model	χ^2^	*df*	RMSEA	CFI	TLI	∆RMSEA	∆CFI	∆TLI	Compared Model
Configural invariance	185.690	108	0.022	0.910	0.890	-	-	-	-
Strong invariance	186.470	118	0.02	0.921	0.911	−0.002	0.011	0.021	1
Strict invariance	190.900	130	0.018	0.929	0.928	−0.002	0.008	0.017	2
Variance-covariance invariance	189.110	131	0.017	0.933	0.932	−0.001	0.004	0.004	3
Latent means invariance	190.450	132	0.017	0.932	0.932	0.000	−0.001	0.000	4

Notes. χ^2^: chi-square; *df*: degrees of freedom; RMSEA: Root Mean Squared Error of Approximation; CFI: Comparative Fit Index; TLI: Tucker-Lewis Index; ∆RMSEA: difference between two RMSEA values; ∆CFI: difference between two CFI values; ∆TLI: difference between two TLI values.

**Table 4:** Prevalence of self-reported adverse health impacts of high heat and humidity conditions according to the adaptation index and age.

Level of Adaptation to Heat	% Who Reported Adverse Health Impacts	Confidence Interval	Coeff. of Variation	Odds Ratio	Confidence Interval	Pr > χ^2^
Individuals who adapt well	45.77	(41.78–49.76)	4.44	1.37	(1.13–1.66)	0.0011
Individuals who do not adapt as well	38.11	(33.66–42.56)	5.95	1.00		
